# Degradation study of lindane by novel strains *Kocuria* sp. DAB-1Y and *Staphylococcus* sp. DAB-1W

**DOI:** 10.1186/s40643-016-0130-8

**Published:** 2016-12-28

**Authors:** Dharmender Kumar, Abhijit Kumar, Jyoti Sharma

**Affiliations:** Department of Biotechnology, Deenbandhu Chhotu Ram University of Science and Technology, Murthal, Sonepat, Haryana 131039 India

**Keywords:** Lindane (hexachlorocyclohexane, HCH), Degradation, 16 S rRNA sequencing, Spray plate assay

## Abstract

**Background:**

This study was carried out to isolate and characterize the bacterial strains from lindane-contaminated soil and they were also assessed for their lindane-degrading potential.

**Methods:**

In this study the enrichment culture method was used for isolation of  lindane degrading bacterial isolates, in which the mineral salt medium (MSM) supplemented with different concentrations of lindane was used. Further, the screening for the potential lindane degrading isolates was done using the spray plate method and colorimetric dechlorinase enzyme assay. The selected isolates were also studied for their growth response under varying range of temperature, pH, and NaCl. The finally selected isolates DAB-1Y and DAB-1W showing best lindane degradation activity was further subjected to biochemical characterization, microscopy, degradation/kinetic study, and 16S rDNA sequencing. The strain identification were performed using the biochemical characterization, microscopy and the species identifies by 16S rDNA sequence of the two isolates using the standard 16S primers, the 16 S rRNA partial sequence was analyzed through BLAST analysis and phylogenetic tree was generated based on UGPMA clustering method using MEGA7 software. This shows the phylogenetic relationship with the related strains. The two isolates of this study were finally characterized as *Kocuria* sp. DAB-1Y and *Staphylococcus* sp. DAB-1W, and their 16S rRNA sequence was submitted to GenBank database with accession numbers, KJ811539 and KX986577, respectively.

**Results:**

Out of the 20 isolates, the isolates DAB-1Y and DAB-1W exhibited best lindane-degrading activity of 94 and 98%, respectively, recorded after 8 days of incubation. The optimum growth was observed at temperature 30 °C, pH 7, and 5% NaCl observed for both isolates. Of the four isomers of hexachlorocyclohexane, isomer α and γ were the fastest degrading isomers, which were degraded up to 86 and 94% by isolates DAB-1Y and up to 93 and 98% by DAB-1W, respectively, reported after 8 days incubation. Isomer β was highly recalcitrant in which maximum 35 and 32% lindane degradation was observed even after 28 days incubation by isolates, DAB-1Y and DAB-1W, respectively. At lower lindane concentrations (1–10 mg/L), specific growth rate increased with increase in lindane concentration, maximum being 0.008 and 0.006/day for DAB-1Y and DAB-1W, respectively. The 16 S rRNA partial sequence of isolate DAB-1Y showed similarity with *Kocuria* sp. by BLAST analysis and was named as *Kocuria* sp. DAB-1Y and DAB-IW with *Staphylococcus* sp. DAB-1W. The 16S rDNA sequence of isolate DAB-1Y and DAB-1W was submitted to online at National Centre of Biotechnology Information (NCBI) with GenBank accession numbers, KJ811539 and KX986577, respectively.

**Conclusions:**

This study has demonstrated that *Kocuria* sp. DAB-1Y and *Staphylococcus* sp. DAB-1W were found efficient in bioremediation of gamma-HCH and can be utilized further for biodegradation of environmental contamination of lindane and can be utilized in bioremediation program.

**Electronic supplementary material:**

The online version of this article (doi:10.1186/s40643-016-0130-8) contains supplementary material, which is available to authorized users.

## Background

Lindane is an organochlorine compound; the ‘γ’ isomer of hexachlorocyclohexane (γ-HCH) primarily used as a fumigant and an insecticide against a wide range of insects. Out of the four isomers of hexachlorocyclohexane (α, β, γ, and δ), ‘γ’ is the only isomer having insecticidal property. Its agricultural use has been banned in most of the developed countries; however, some developing countries are continuing its use due to economic factors such as low cost (Johri et al. 1998). Due to its continuous use throughout the world, lindane-contaminated sites are prominent worldwide. Once HCH enters the environment, it can distribute globally (Simonich and Hitéis 1995) and can also persist in various environments (Abhilash 2009; Abhilash et al. 2008). The various sites of lindane contamination have been reported in different countries, viz., Europe (Concha-Grana, et al. 2006), America (Osterreicher-Cunha et al. 2003; Phillips et al. 2006), and Asia (Prakash et al. 2004; Zhu et al. 2005). The half-life period reported for lindane in soil and water are 708 and 2292 days, respectively (Beyer and Matthies 2001). Lindane causes various environmental impacts and persists in the soil for long periods. Therefore, toxicity and threats of environmental contamination are of great concern, and this problem can be solved through biodegradation-based approaches. The need of the hour is to develop procedures that could remove these toxic compounds by converting them to non-toxic form intermediate simple compounds. The various approaches of decontamination of HCH like chemical treatment, incineration, and land filling available, but they lack widespread application due to their cost factor and toxicity concerns to the living system. The bioremediation technology has been proposed as a promising tool for in situ detoxification of pesticide-contaminated sites. The various soil microorganisms capable of degrading and utilizing the organochlorine γ-hexachlorocyclohexane as a source of carbon have been reported over the last two decades at various places (Sahu et al. 1990; Adhya et al. 1996; Okeke et al. 2002; Nawab et al. 2003). Although the use of HCH has been banned in most of the countries, γ-HCH and its non-insecticidal isomers α, β, and δ still continue to pose environmental and health hazard (Pavilikova et al. 2012).

A variety of pesticides are being used in agriculture crops for the control of various insects. In spite of their agricultural benefits, pesticides are often considered a serious threat to the environment because of their persistence in environment for long period of time. Therefore the removal of pesticides from the environment source and ecological site is a topic of research interest for the scientists worldwide. In recent years, the use of degrading microorganisms or removing pesticides has been employed as the ecofriendly approach. This will enrich the in situ degradation and ex situ degradation as well. Therefore, the microbial bioresource has the great potential in solving current environmental problems. The bioremediation-based approaches possess high efficiency, sustainability, and their ecofriendly nature provides a solution to traditional physico-chemical remediation. Shrivastava et al. (2015) characterized a novel LinA type 3 δ-hexachlorocyclohexane dehydrochlorinase. The *LinA* gene involved the synthesis of first enzyme of the microbial degradation pathway of lindane, and leads to the dehydrochlorination of all four HCH isomers except beta-isomer. The two variants, LinA type 1 and LinA type 2, differ at 10 out of 156 amino acid residues. This study describes the characterization of a new variant of this enzyme, LinA type 3 gene was identified from a HCH-contaminated soil sample using metagenomic approach. Sun et al. (2015) conducted four pilot-scale test microcosms bioaugmentation study for the remediation of organochlorine pesticides (OCPs)-contaminated soil. They noted the effects on degradation of HCHs and dichlorodiphenyltrichloroethanes (DDTs) and found that nutrients/plant bioaugmentation enhanced the degradation of 81.18 and 85.4%, respectively). In order to develop the cleanup strategy of the polluted sites, recent study conducted by Laquitaine et al. (2016) has demonstrated the biodegradability of HCH in agricultural soils from Guadeloupe (French West Indies) and conducted studies which lead to the identification of the degrading genes among the characterized strains. The chlorinated pesticides viz. HCH, chlordecone and dieldrin, were used in agriculture until the start of 1990, this resulted in a contamination of the soil and water in the areas of banana production. They have conducted studies for lindane degradation in soil slurry microcosms. The 40% lindane degradation efficiency was reported in 30-day treatment experiment. During the course of this degradation study, the lindane concentration decreased from 6000 to 1330 and 800 to 340 ng/mL for the biotic and abiotic soils samples, respectively. Molecular studies for gene analysis indicating that HCH degradation was probably mediated by bacteria closely related to *Sphingomonadaceae* family bacteria.

Considering, the adverse environmental impacts of lindane and its toxicity, in the present study, we have attempted to isolate and characterize novel bacterial strains from lindane-contaminated soil collected from Lucknow, India. The enrichment culture method was used for the screening of the isolates using mineral salt medium supplemented with varying concentration of lindane as sole carbon source. Also, we have tried to study the kinetics of these two potent bacterial strains capable of degrading γ-HCH grown in the shake flask culture. The isolation and characterization of the strains from the ecological habits lead to find out the new genes and enzymes involved in the degradation of toxic compound by the activity of the microbial strains. Further new strains will enrich the gene pool for the biodegradation of lindane/or other toxic compounds present in the environment. These strains may be utilized as the microbial source for the degradative gene(s), and these genes can be transferred to non-degradative strains by recombinant DNA technology.

## Methods

### Chemicals, soil sampling, and isolation of bacterial strains

The technical grade HCH isomers (α, β, γ, and δ; 99.9% pure) were purchased from Sigma-Aldrich (St. Louis, MO, USA), and other chemicals and reagents of technical/molecular grade were obtained from Qualichem, Ranbaxy etc. The media and molecular biology kits were obtained from Hi-Media, Mumbai India. Soil samples were collected in sterile polythene bags from a contaminated site of Lucknow, UP, India. The collected soil samples were brought to the laboratory, dried, and kept at 4 °C until further microbial isolation was performed. The pH value of the soil samples ranged between 6.5 and 7.8.

The isolation of bacterial strains was carried out from soil samples using enrichment culture technique (Dams et al. 2007). Mineral salt medium (MSM) was prepared in 250 mL Erlenmeyer flask containing (per liter) potassium dihydrogen phosphate, 0.85 g; dipotassium hydrogen phosphate, 2.17 g; disodium hydrogen phosphate, 3.34 g; ammonium chloride, 0.1 g; magnesium sulfate, 0.5 g; calcium chloride, 0.5 g; ferrous sulfate, 0.01 g; sodium molybdate, 0.01 g; at a pH of 7.2 ± 0 5 (Sahu et al. 1990). Two grams of collected soil sample and 10 mg/L of lindane was weighed and added to 100 mL of sterile MSM broth. The stock solutions of the HCH were filter sterilized (0.22 micron Millipore syringe filter) and then aseptically transferred to autoclaved cooled MSM. After thorough mixing, the flask was incubated at 30 °C for 7 days in a rotary shaker at 120 rpm. Subsequently, 1 mL of the inoculum having a cell density of approximately 5 × 10^3^ CFU/mL was taken from the flasks with a micropipette and was transferred to sterile medium (100 mL) containing the same amount of lindane concentration. The inoculum was transferred to fresh media each time with increasing lindane concentration from 10 to 100 mg/L in a stepwise manner. After acclimatization of the culture, the serial dilution up to 10^−4^ dilution was spread plated and used for isolation of bacterial colonies on mineral agar plates supplemented with 10 mg/L of lindane. The plates were incubated under aerobic conditions at 30 °C in incubator (Remi Instruments, India). After 24 h, colonies with unique morphology were sub-cultured on fresh agar plates in the form of single culture and preserved at 4 °C till further use.

### Screening and selection of lindane-degrading bacteria (quantitative study)

The bacterial isolation was done using the enrichment culture technique and tested qualitatively for lindane-degrading activity by spray plate dehalogenase assay (Phillips et al. 2001; Manickam et al. 2008). Spray plates were prepared with MSM supplemented with 1.5% agar in petri dishes, and after solidification of the plates, the pure culture was streaked on the surface of these plates. The 0.5% of lindane solution was prepared by dissolving lindane powder in acetone and was sprayed on the surface of preset agar plates. These plates were further incubated for seven days at 28 ± 2 °C in incubator. This method was used for the isolation of lindane-degrading bacteria RP-1, RP-2 and RP-3 reported in our other study (Pannu and Kumar 2014).

The isolates showing best lindane-degrading activity in spray plate method viz. DAB-1Y and DAB-1W was selected from 20 isolates and they was further subjected to analysis by argentometric method given by Greenberg et al. (1992) for quantitative degradation studies conducted in shake flask. It gives estimation of chloride ion released into the medium by the lindane-degrading strains due to activity of dechlorinase enzyme. The isolates were inoculated in MSM broth supplemented with 10 mg/L of lindane and incubated at 30 °C at 120 rpm in orbital shaker. The 1 mL of culture was withdrawn at different time periods viz 2, 4, 6, 8, and 28 days. The *Sphingobium japonicum* MTCC 6362 was procured from Microbial Type Culture Collection (MTCC), Institute of Microbial Technology (IMTECH), Chandigarh, India, and this was used as reference strain in all the experiments as positive control. The biodegradation potential of the finally selected isolates was evaluated by analyzing the residual lindane in the medium which was calculated using the following formula:

$${\rm Residual}\,\, {\rm lindane}\,(\%)= (C_t /C_0) \times 100$$

where *C*_0_ = initial concentration of lindane in the medium; *C*_*t*_ = lindane concentration at time t.

### Kinetic study for HCH degradation

The kinetic study of degradation mediated by the reported isolates DAB-1Y and DAB-1W, in batch culture, was carried out by the method described by Lodha et al. (2007) with slight modification. The whole cells from the culture grown in MSM broth were used as source enzyme released in the culture supernatant and used to study γ-HCH degradation. The cell biomass was obtained by growing the bacterial culture in minimal mineral media supplemented with 10 mg/L of HCH in the incubator at 30 °C for 8 to 28 days. The cells were harvested at 4 °C by centrifugation, suspended with minimal mineral media, and then used for biodegradation assay for the determination of degradation γ-HCH by the dechlorinase activity. The kinetics of the growth of the microorganisms in shake flask batch culture study was carried out to know the degradation under different time intervals. Therefore, in this study, kinetics of the bath culture was carried out for the degradation of γ-HCH by the following equations. The cell growth kinetics in a batch reactor may be modeled by following Eq. (1):

1 $$\cdot\frac{{{\text{d}}X}}{{{\text{d}}t}} = \mu_{\text{g}} X-K_{\text{d}} X = \mu_{\text{net}} X   ...................................$$

For substrate,

2 $$\frac{{{\text{d}}S}}{{{\text{d}}t}}= -1/Y({\text{d}}X/{\text{d}}T)..........................................$$

*μ*_g_ is a function of *S*.

For non-toxic compounds, Monod’s equation as given below is generally applicable:

3 $$\mu_{\text{g}} = \mu_{ {\rm max} } \;\frac{S}{S + K}..................................................$$

During exponential phase. Equation (1) reduces to following equation:

4 $$\frac{dx}{dt}\; = \;\mu_{\text{g}} X.............................................$$

This is because *K*_d_ is neglected during exponential phase.

During initial phase, *S* may be taken equal to *S*_0_. Therefore:

5 $$\ln \frac{x}{{x{}_{0}}}\; = \;\mu_{\text{g}} t............................................................................$$

where *S* is the concentration of substrate (mg/L); *S*_*0*_ the initial concentration of substrate (mg/L); *T*, t the time (days); *X* the concentration of biomass (mg/L); *X*_*0*_ the initial concentration of biomass (mg/L); *Y* the observed yield coefficient; *μ*_g_ the specific growth rate (h − 1); *μ*_max_ the maximum specific growth rate (h − 1); *μ*_net_ the net specific growth rate (h − 1); and *K* is the rate constant for bioisomerization (day − 1).

The kinetic study was performed for γ-HCH degradation for initial concentrations 1, 2, 5, 10, 15, 20, 30, 40, and 50 mg/L in different flasks and two control experiments (without biomass and without substrate) were carried out for each concentration. The samples at particular time interval were withdrawn from the flasks from shaker and analyzed for the bacterial growth by monitoring the optical density of the culture at 600 nm using UV–Vis spectrophotometer (Shimadzu, Japan).

### Effect of physiological parameters on lindane biodegradation

Different physiological parameters like temperature (20–50 °C), pH (3–11), NaCl (1–5%), and incubation time (5–20 days) were optimized to study their effect on biodegradation by isolates DAB-1Y and DAB-1W. The *Sphingobium japonicum* (MTCC 6362) was used as reference control in all the experiments. Degradation study was carried out in mineral salt medium (MSM) containing lindane (10 mg/L) as sole carbon source and analyzed by varying one parameter at a time, keeping others constant. The culture showing growth, by measuring OD_660_ using UV–Vis spectrophotometer was taken as indicator of lindane utilization. The maximum value of OD_660_ values obtained was taken as optimized set of parameters for lindane degradation (data not shown).

### Strain identification

The finally selected isolates, DAB-1Y and DAB-1W, were characterized by morphological and biochemical characterization; 16S rRNA sequencing and one isolate (DAB-1W) in addition to 16S rRNA were also characterized by GC-FAME technique. The former technique is based on the sequencing using universal primers to the 16S rDNA of our isolates and later is based on the analysis of the fatty acid profile of the bacteria. As the fatty acid profile of the bacterial species is the unique signature to be helpful in detection on the bacterial species from the varied samples. The fatty acid is also produced by the bacteria under specific media supplementation and studied by Ehrhardt et al. (2010), but in our study, the identification is done based on the fatty acid which was produced in the customized medium and experiment was conducted in duplicate along with control. Therefore, the fatty acid uniquely produced during our study was characterized and detected through FAME, and the identification was done using Sherlock identification system which identifies fatty acids from our sample from the screening of fatty acid library. The fatty acid profile identifies the microbial species. A variety of microorganisms degrade the HCH isomers and their degradation studied by many researchers (reviewed by Alvarez et al. 2012).

The identification of the bacteria involved in degradation was proceeded mainly by 16S rRNA sequencing for strain identifications, but one isolate was also characterized by GC-FAME analysis as well. The 16S rRNA is approx 1500 bp region of genomic region of bacteria and considered as genetic signature for the identification of the bacteria. A number of studies were conducted based on this technique, and it was considered that 16S region is conserved during the course of evolution among bacterial linage. Therefore, it helps microbiologists and pathologists in the characterization of new strain isolated from various sources. Similarly, GC-FAME-based identification was also considered rapid and reliable technique for the identification of microorganisms based on the pattern of cellular fatty acid in bacteria, which is unique signature and aids in their identification and classification. The only commercial available database for the identification by fatty acid methyl ester (FAME) analysis is Sherlock microbial identification system (MIS) developed by Microbial ID, Inc (MIDI). This was developed by Sasser et al. (2001) for the identification of aerobic bacteria. Therefore, it represents rapid, accurate, less expensive approach for the identification of more than 1500 microbial species (www.midi.com/pages).

#### Morphological and biochemical characterization

The different morphological studies of the selected lindane-degrading isolates, DAB-1Y and DAB-1Y, were carried out to determine their cell shape, cell size, color, cell motility, capsule formation, colony morphology, pigmentation etc., by growing the culture on nutrient agar plates followed by preliminary identification using Gram’s staining. The isolates were further identified up to genus level by biochemical tests, 16S rRNA sequencing, and GC-FAME analysis.

The isolates, DAB-1Y and DAB-1W, were identified biochemically according to the method given by Karn et al. (2011) in which *Kocuria* sp. CL2 showed similar biochemical results with respect to colony shape, cell grouping starch and casein hydrolysis, gelatin liquefaction, methyl red and vogues–proskauer test, catalase test, adonitol, sorbitol, citrate, ornithine, lysine and urea utilization, nitrate reduction, phenylalanine deamination, triple sugar iron test, H_2_S production, and various carbohydrate fermentation tests like mannitol, galactose, xylose, rhamnose, sucrose, glucose, arabinose and lactose) with the standard protocols given in Bergey’s Manual of Determinative Microbiology (Holt et al. 1994). The KB10 kit (Hi-Media, Mumbai, India) was used for biochemical characterization and other biochemical tests were carried out using standard methods (Cappuccino and Sherman 2010). The gelatin liquefaction test was performed by method given by Clarke (1953).

### Molecular characterization of isolates

#### Genomic DNA extraction

Total DNA from the bacterial isolates was isolated using the Alkaline lysis method as described by Kate Wilson (1987). In each case, 5 mL of an overnight culture was centrifuged at 5590×*g* for 5 min and the resulting pellet was resuspended in 574 μL of TE (10 mM Tris, 1 mM EDTA, pH 8) buffer. Into this, 300 μL of cell lysis buffer containing lysozyme, SDS, CTAB, and proteinase-K was added and the mixture was incubated for 1 h at 37 °C. Subsequently, DNA was separated from the cell lysate using phenol/chloroform/isoamyl alcohol (25:24:1) and chloroform/isoamyl alcohol (24:1). By the adding of isopropyl alcohol, the DNA was precipitated and the pellet was resuspended in 50 μL of TE buffer. Then RNase was added to remove RNA before storage of the samples at −20 °C. The quality of the extracted DNA was analyzed by electrophoresis in 1% agarose gel supplemented with ethidium bromide (10 mg/mL). The DNA bands were visualized in UV-gel documentation system (BioRad, USA).

#### PCR amplification of 16S rDNA

The polymerase chain reaction (PCR) amplification for targeting the 16S rDNA from the genomic region of isolate DAB-1Y was carried out using 8F (5′-AGAGTTTGATCMTGGCTCAG-3′) and U1492R (5′-GGTTACCTTGTTACGACTT-3′) forward and reverse primers, respectively. Similarly, the isolate DAB-1W DNA was amplified through PCR using 27F (5′-AGAGTTTGATCMTGGCTCAG-3′) and 1492R (5′-TACGGYTACCTTGTTACGACTT-3′) primer(s), respectively. Two µL of purified genomic DNA was used as template. The 16S rRNA gene was amplified using GeneAmp 2700 PCR systems (BioRad, USA). The reaction mixture for the PCR contained 4 µL of 10× PCR buffer with MgCl_2_; 10 mM of dNTPs; 0.5 µL of each primer; 10 mM and 0.5 µL of 5 U/µL Taq DNA polymerase (Sigma, USA) in a final volume of 25 µL. The PCR was performed with an initial denaturation at 92 °C for 2 min followed by 35 cycles of 92 °C for 1 min, 48 °C for 30 s, and 72 °C for 2 min, and a final extension of 72 °C for 6 min was given. The PCR products were separated by electrophoresis on a 1% agarose gel and visualized under gel documentation system after staining with ethidium bromide.

#### Sequencing and phylogenetic analysis

The amplified PCR products were sent for sequencing to Yaazh Xenomics, Chennai, India. The 16S rDNA sequence obtained checked for any chimeric sequence using online search-based DECIPHER bioinformatics tool (Wright et al. 2012). The comparison of the sequences obtained was done with the GenBank database using Basic Local Alignment Search Tool (BLAST)N program using National Centre of Biotechnology Information (NCBI) database (http://blast.ncbi.nlm.nih.gov/Blast.cgi), and similar sequences were downloaded and aligned with Muscle; a phylogenetic tree was constructed using UGPMA clustering method using MEGA7 software by Neighbor-joining method (Kumar et al. 2016).

#### 16S rRNA accession numbers

The two PCR product amplified by the 16S rRNA universal primers in this work was deposited to NCBI GenBank with accession numbers KJ811539 and KX986577, respectively.

#### Identification of strain by GC-FAME

Gas chromatography-fatty acid methyl ester (GC-FAME) is an alternative tool to classical microbiological identification techniques. The genomic expressions (i.e., DNA/RNA homology, lipid composition, protein pattern etc.) are conserved among a group of microbial species and they seem to be the reliable indicators for the identification. This analysis makes use of short chain fatty acids to characterize genera and species of bacteria. As compared to FAME, 16S rRNA-based species characterization is more reliable technique because it is molecular biology-based technique. The 40 mg of pre grown bacterial cells was harvested and saponified. After this, the fatty acids were extracted and methylated to form fatty acid methyl esters (FAME). The samples were sent to Royal Life Science, Hyderabad, India; for GC-FAME analysis. These fatty acids from the sample were analyzed using gas chromatography with the help of MIDI Sherlock software for FAME pattern analysis. The aerobic library (RTBSA 6.21) was referred for analysis of the DAB-1W strain and was performed as per the method given by Sasser (2001).

## Results and discussion

The isolates DAB-1Y and DAB-1W were found potential HCH degrading strains and seem to have great potential for the gamma-HCH degradation program. The results and discussion based on their characterization are presented below based on biochemical, microscopy, degradation analysis, and molecular identification of their species based on molecular (16S rRNA sequencing and also identification by the GC-FAME.

### Enrichment, isolation, and screening of lindane-degrading bacteria

**Fig. 1:**
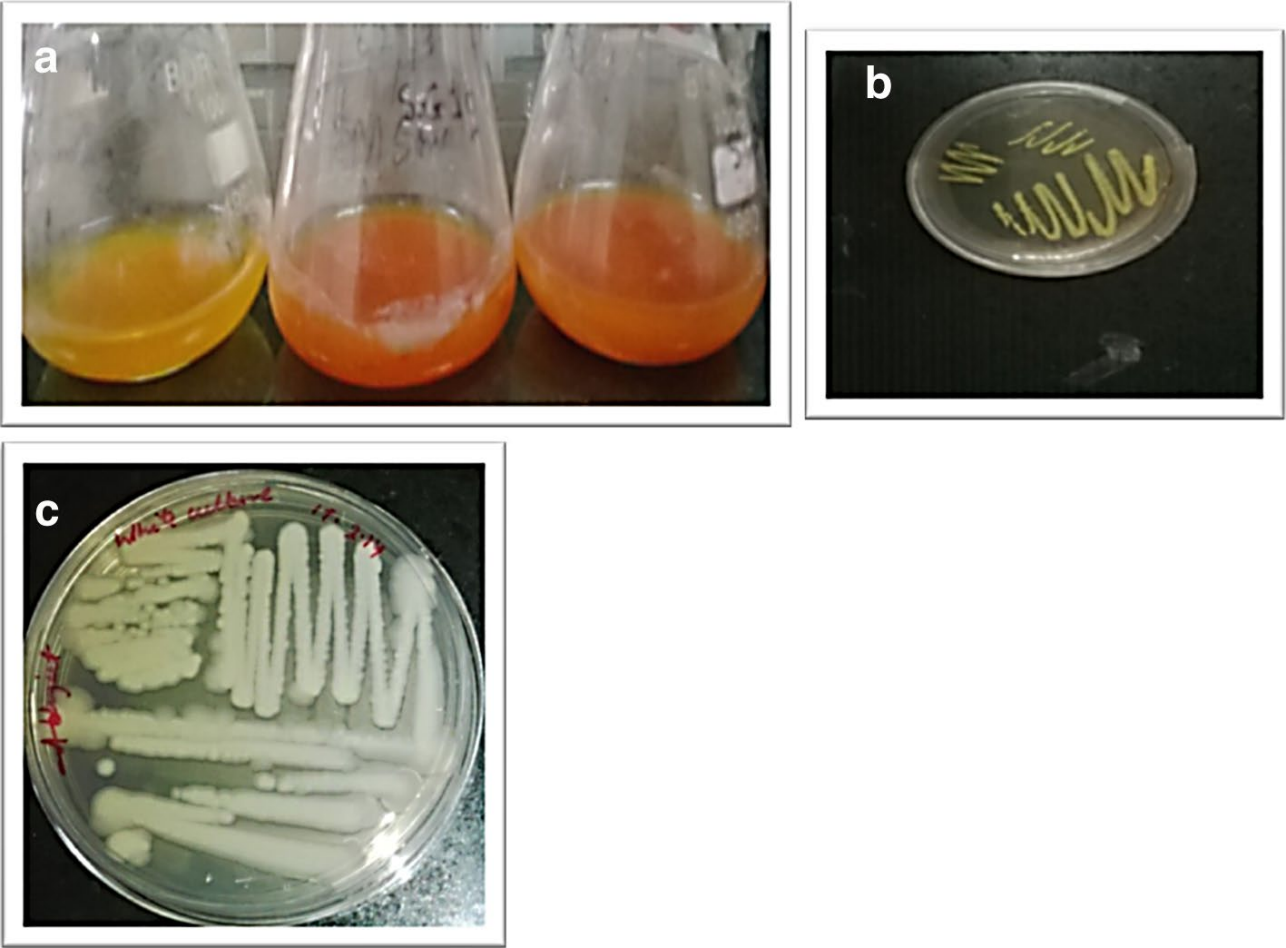
**a** Flasks showing lindane-degrading activity based on chloride ion release titration-based assay, I flask negative, II & III showing positive results (from *left* to *right*); **b** isolate DAB-1Y; **c** isolate DAB-1W

For the isolation of lindane-degrading bacteria, soil samples were collected from Lucknow, India, which had a long history of HCH production and application. Soil cores (0–20 cm) taken from selected spots were collected in sterile plastic bags and stored at 4 °C until microbial isolation. Bacterial isolation was carried out from soil samples using enrichment culture technique (Dams et al. 2007). Two grams of collected soil sample and 10 mg/L of lindane was weighed and added to 100 mL of sterile mineral salt medium. After thoroughly mixing the media components, the flask was incubated at 30 °C for 7 days in rotary shaker at 120 rpm. Subsequently 1 mL of the inoculum having approximately 5 × 10^3^ CFU/mL cell density from the flasks was transferred into sterile medium (100 mL) containing supplementation of same lindane concentration. The inocula were transferred to fresh media each time with increasing lindane concentration from 10 to 100 mg/L in a stepwise manner. After acclimatization, serial dilution from 10^−2^ to 10^−4^ dilution and spread plate method on MSM plates supplemented with 10 mg/L lindane were used for isolation of bacterial colonies giving 55, 43, and 33 cfu/mL, respectively. Colonies were sub-cultured on fresh agar plates in the form of single culture and preserved at 4 °C in refrigerator. The isolates DAB-1Y and DAB-1W were identified based on their cultural characteristics and microscopic features. On plate streaking and observation after incubation overnight at 30 °C, the culture shows typical yellow and white morphology reported for isolates DAB-1Y and DAB-1W, respectively (Fig. 1a, b). The microscopic examination of Gram’s stained culture preparation of DAB-1Y as rods in chains and DAB-1W as small *cocci* bacteria (data not shown).

The selected bacterial isolates were tested qualitatively for lindane-degrading activity using spray plate method. The formation of lindane clearance zone surrounding bacterial colonies indicated the utilization of lindane. The chloride ions released during the degradation study were analyzed based on the titration-based assay, and the change of color from yellow to pink is observed (Fig. 1c) Similar lindane degradation zones were observed for bacterium *Pseudomonas paucimobilis* (Senoo and Wada 1989), fungus *Conidiobolus* 03-1-56 by (Nagpal et al. 2008), and for yeast *Rhodotorula* sp. VITJzNo3 (Salam et al. 2013). The clear halo zones also appeared on agar plates containing precipitated γ-HCH around colonies of lindane-degrading isolates reported by Thomas et al. (1996). The production of halo zones around culture growth observed after incubation leads to the conclusion that the enzymes involved in γ-HCH dechlorination are extracellularly produced and the secretion of these enzymes from the bacterial culture leads to the production of clear haloes around colony.

### Degradation study of lindane (quantitative analysis of chloride ion release)

**Fig. 2:**
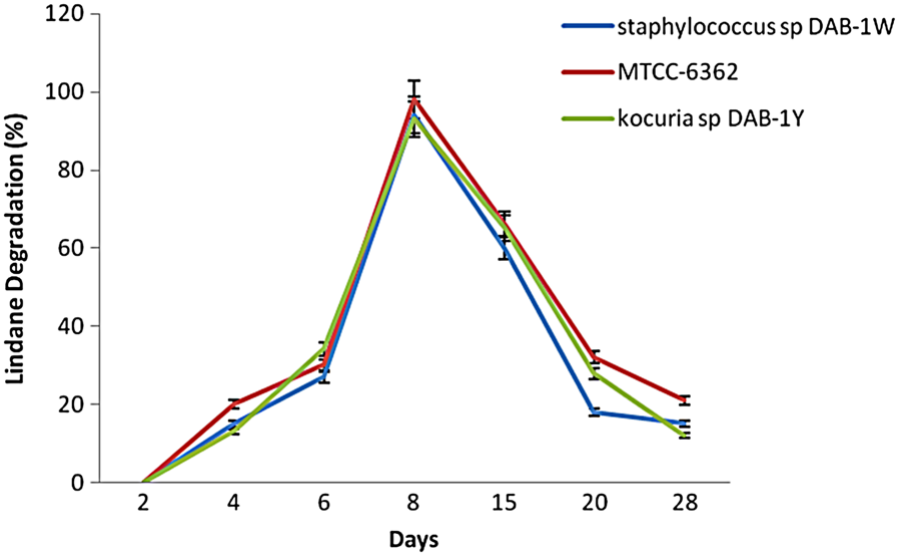
Degradation of γ HCH observed at different time intervals for DAB-1Y and DAB-1W along with standard strain MTCC 6362 (*bar* indicates standard error at 5% value)

When lindane was used as the sole carbon source and supplemented with MSM broth, the isolates utilize and their growth was observed in the form of turbidity of the medium. The extent of mineralization of lindane was determined by quantifying the release of inorganic chloride ions and analyzing the percent residual lindane in the medium. The γ-HCH degrading efficiency increased from 13 to 94% and from 15 to 98% with increase in time of incubation from 2 days to 8 and up to 28 days, for isolates DAB-1Y and DAB-1W, respectively (Fig. 2). The results were comparable to standard lindane-degrading strain MTCC6362 which showed 20% lindane degradation in 2 days and 98% degradation in 8 days. Similar results have been reported by Gupta et al. (2000). Our earlier study demonstrated that the isolates RP-1 and RP-3 showed 69.5 and 65% lindane degradation after 10 days of inoculation, respectively, and isolate RP-9 was able to degrade 62% of lindane after 15 days of inoculation (Pannu and Kumar 2014). The various studies for the isolation of novel lindane-degrading strains have been reported by other studies and their tolerance to lindane presented for comparison with our strains reported in this study (Table 1). It was seen that our two reported isolates DAB-1Y and DAB-1W were potential lindane degrading, and further these strains were characterized for strain identification.

**Table 1:** Comparison of lindane degradation by the bacterial strains reported in different studies

Sr. No.	Bacterial strain reported	HCH-tolerance	Reference
1.	*Kocuria* sp. DAB-IY and *Staphylococcus* sp. DAB-1W	100 mg/L	This study
2.	*Streptomyces* sp. M7	16.6 mg/L	Sineli et al. (2016)
3.	*Sphingomonas baderi* sp.	100 mg/L	Kaur et al. (2013)
4.	*Arthrobacter florescens* and *Arthrobacter giacomelloi*	100 mg/L	De Polis et al. (2013)
5.	*Actinobacteria* sp. and *Streptomyces* sp.	1.66 mg/L optimum for degradation	Fuentes et al. (2010, 2011)
6.	*Kocuria rhizophila, Microbacterium resistens, Staphylococcus equorum, Staphylococcus cohnii subsp.ureolyticus*	5–100 μg/mL	Abhilash et al. (2011)
7.	Lindane-degrading consortia	300 μg/mL after 108 h	Elcey and Kunhi (2010)
8.	*Azotobacter chroococcum* JL102	100 ppm	Anupama and Paul (2010)
9.	*Sphingomonas* sp. NM05	100 μg/mL	Manickam et al. (2008)
10.	*Sphingomonas* sp. NM05	100 μg/mL	Manickam et al. (2008)
11.	*Pseudomonas aerogenosa*	1–10 mg/L	Lodha et al. (2007)
12.	*Pseudomonas aerogenosa* ITRC5	5 mg/mL soil	Kumar et al. (2006)
13.	*Pandoraea sp*.LIN-3	100 mg/L	Okeke et al. (2002)
14.	*Bacillus brevis* and *Bacillus circulans*	5 μg/mL	Gupta et al. (2000)
15.	*Arthobacter citreus* BI-100	100 mg/L	Datta et al. (2000)

### Effect of different physiological parameters on lindane degradation

**Fig. 3:**
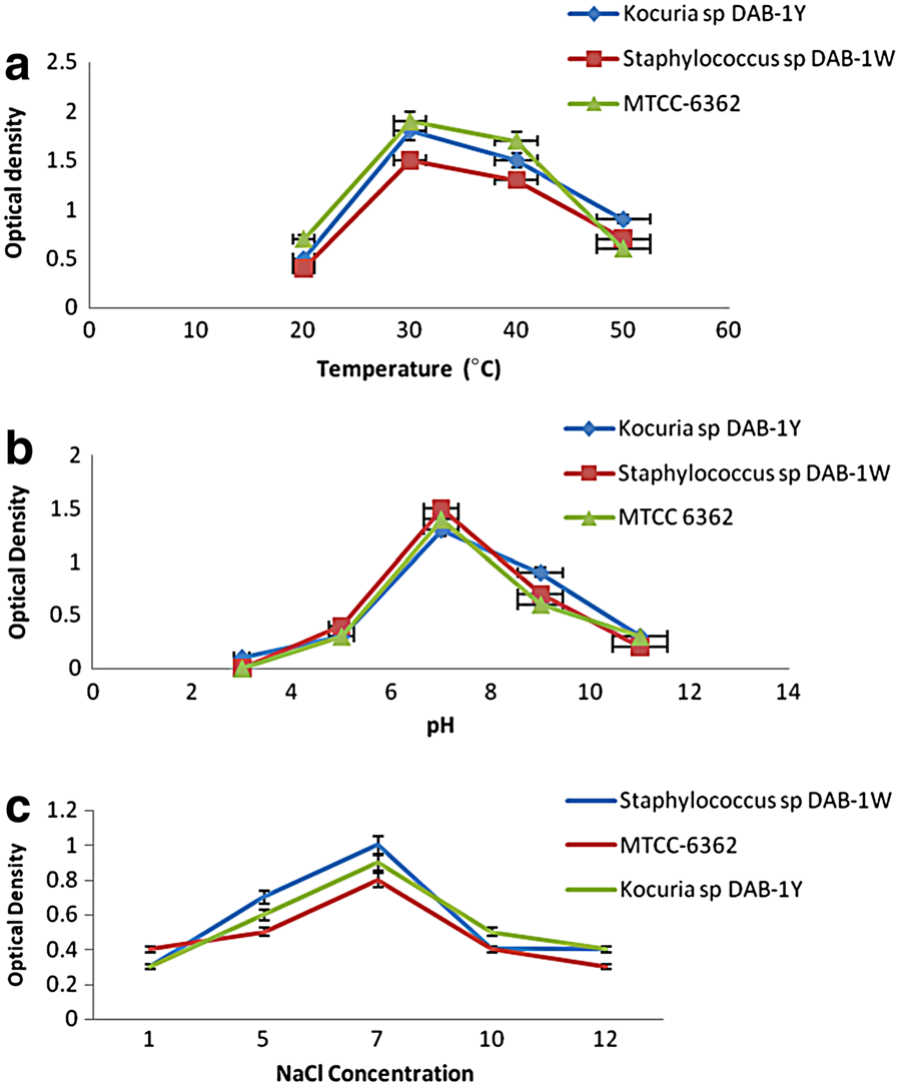
Effect of different physiological parameters on lindane degradation for *Kocuria* sp. DAB-1Y, *Staphylococcus sp* DAB-1W and standard strain MTCC 6362 by **a** temperature, **b** pH; **c** NaCl (*bar* *indicates Standard Error at 5% value*)

To determine the effect of various physiological parameters (temperature, pH, NaCl) on lindane degradation, the strains were incubated at different temperatures varying from (20, 30, 40, and 50 °C), pH varying from pH (3 to pH 11), and NaCl (1–12%). The isolates DAB-1Y and DAB-1W showed similar patterns of growth in lindane-containing medium. The degradation was observed over a broad range of temperature and pH range indicating that microbial remediation was possible over a broad range of environmental conditions. The optimum growth was observed at 30 °C (Fig. 3a), pH 7 (Fig. 3b) and 5% NaCl (Fig. 3c) for our two isolates along with one strain MTCC6362 reported in this study. Beyond 30 °C and pH 7, bacterial growth started decreasing and slowest growth was observed at the two extreme temperature and pH values. Broad ranges were observed by other researchers also for pesticides degradation. Other workers have also reported that for lindane degradation, a neutral range of pH 6–8 was most favorable (Elcey and Kunhi 2010). Similar results have also been reported by Okeke et al. (2002) who reported pH 9 as optimum for growth and biodegradation of α and γ isomers of lindane. Another strain of *Clostridium rectum* was reported by (Ohisa and Yamaguchi 1978), which degraded lindane optimally at pH 7–8 in anaerobic pure culture studies.

The incubation temperature of 30 °C has been found as optimum temperature for lindane degradation (Zhang et al. 2010, 2012; Salam et al. 2013). However, rapid degradation of lindane by *Sphingobium* strains has been reported by Zheng et al. (2011) even at low temperature (4 °C) indicating that lindane degradation could be achieved even in colder contaminated regions. We have also tested the degradation of the inoculated strains in the liquid culture at 4 °C and checking the dechlorinase activity of the culture filtrate received after centrifugation. No activity was observed in DAB-1W and DAB-1Y strains. The percentage lindane degradation was studied for different incubation times ranging from 2 to 8 days. It was observed that lindane degradation increased with increase in incubation time from 2 to 28 days (Fig. 2). It was 13, 15, and 20% reported after 2 days for DAB-1Y, DAB-1W, and MTCC 6362, respectively. The degradation increased to 24, 27, and 30% after 4 days and increased to 38, 47, and 60% after 6 days for DAB-1Y, DAB-1W, and MTCC 6362, respectively. The maximum lindane degradation was observed 98% for MTCC 6362 after 8 days incubation. Our isolates DAB-1Y and DAB-1W demonstrated comparable results to standard strains and achieved 94 and 98% degradation, respectively, after 8 days of incubation under standard conditions.

Also, the four isomers of HCH were analyzed separately for their degradation behavior, and degradation was carried out for 2-day interval for α, γ, and δ isomers and at 7-day interval for β HCH for 8 and 28 days, respectively. Table 2 shows that isomers α, γ, and δ were degraded successfully by our strains. All the isomers, except β HCH, showed more than 80% degradation after 8 days of inoculation. Isomer β was the most recalcitrant isomer (Kaushik 1989) and was thus analyzed for a total period of 28 days at 7-day interval. However, the maximum 35 and 32% lindane degradation only could be observed even after 28 days of incubation by isolates DAB-1Y and DAB-1W, respectively. The isomers, α and γ, were the fast degrading isomers that degraded up to 86 and 94% by DAB-1Y and up to 93 and 98% by DAB-1W, respectively, after 8 days of incubation. It was observed in this study that after 28 days of incubation, the degradation all four HCH isomers decreased due to inhibition of enzyme in the shake flask after long treatment of incubation. Therefore, 8 days of incubation period was found to be the efficient degradation of gamma-HCH in liquid medium. Similar results were reported by Kaushik (1991) where the isomers α and γ were found to be the fastest degrading isomers and degraded from 81 to 95% and from 95 to 100%, respectively, after 8 days. Isolates DAB-1Y and DAB-1W were also capable of degrading δ HCH up to 80 and 76%, respectively. It has been reported in earlier studies that *Pseudomonas* sp. (Sahu et al. 1992) and *Bacillus brevis and Bacillus circulans* (Gupta et al. 2000) also degrade the four isomers of HCH. Also, earlier studies indicate that aerobic conditions are more favorable for HCH degradation as reported by Bachmann et al. (1988) and Tu (1976) as compared to anaerobic conditions (MacRae et al. 1967; Castro and Yoshida 1974). Bacterial strain *Kocuria* sp. CL2 reported by Karn et al. (2011) was able to grow and remove Pentachlorophenol more than 87% in 600 mg/L indicating the efficiency of *Kocuria* sp. CL2 to remove PCP at higher concentrations. Thus, strains reported in our study can also be used for bioremediation of lindane-contaminated agricultural sites.

**Table 2:** Morphological and biochemical characterization of isolates DAB-1Y and DAB-1W

Characters	Observation		Characters	Observation	
Morphological	Isolates		Biochemical characteristics	Isolates	
	DAB-1Y	DAB-1W		DAB-1Y	DAB-1W
Cell shape	Rods in chains	Rods	Citrate utilization	+	−
Gram straining	+	+	Lysine	−	+
Colony morphology on NA plates	Yellow smooth	White smooth	Ornithine	−	+
Pigmentation	Yes	No	Urease	+	−
			TDA	−	+
Physiological parameters			Nitrate reduction	−	+
Growth at 4 °C	NA	No	H_2_S-production	−	+
Growth at 8 °C	Y	No	Glucose	+	+
Growth at 25 °C	Y	Y	Adonitol	−	−
Growth at 45 °C	Y	Y	Lactose	−	−
Growth with 1% NaCl	Y	Y	Arabinose	−	+
Growth with 5% NaCl	YYY	Y	Sorbitol	−	+
Growth with 7% NaCl	Y	Y	Starch hydrolysis	Y	N
Growth with 10% NaCl	Y	No	Cell motility (by hanging drop method)	Y	N
Growth with 12% NaCl	No	No	Gelatin hydrolysis	Y	N
Growth at pH 2	No	No			
Growth at pH 4	No	No			
Growth at pH 5	YYY	Y			
Growth at pH 7	Y	Y			
Growth at pH 8	Y	Y			
Growth at pH 9	No	Y			
Growth at pH 11	No	No			

### Kinetics of HCH degradation

**Fig. 4:**
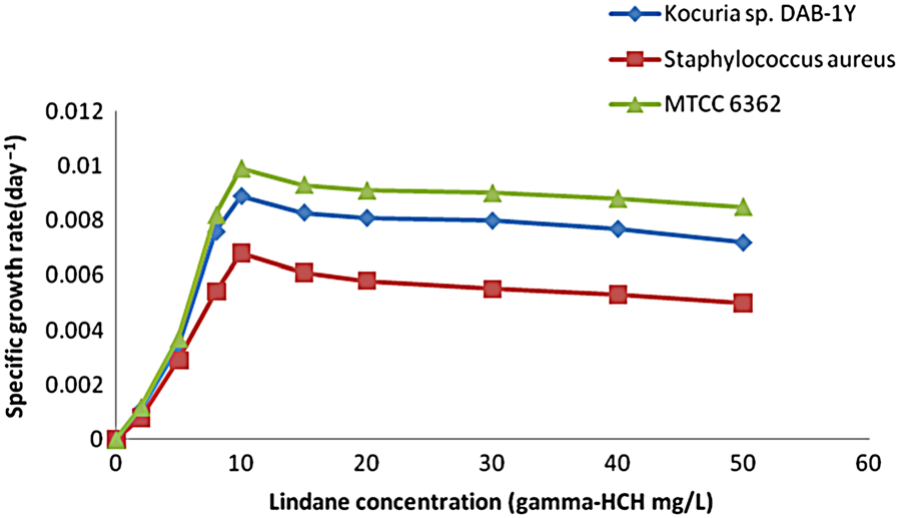
Effect of initial γ-HCH concentration on the specific growth rate of *Kocuria sp.* DAB-1Y and *Staphylococcus* sp. DAB-1 W

To design large-scale bioreactors successfully for bioremediation of contaminants, it is mandatory to accurately predict the growth parameters of microorganisms in lab study based on the shake flask degradation experiments. This will ease the optimization at large-scale growth of the microorganism. Also, mathematical models that are used should not be too complex in order to reduce the experimental work needed to determine kinetic parameters (Chung et al. 2005). Earlier reports have reported the effect of biokinetic parameters on degradation using different microorganisms involved in the degradation of toxic organic compounds, and biomass growth data (cfu/mL) from different initial HCH degradation batch experiments were plotted on a semi logarithmic graph (Kumar et al. 2005; Park et al. 2002; Nuhoglu and Yalchin 2005; Sa and Boaventura 2001). Lodha et al. (2007) have reported biokinetics of HCH degrading pure aerobic cultures. Hence, we carried out studies to evaluate growth kinetics of HCH degrading strains. It was observed that after a short lag phase, linear plots were obtained at all initial concentrations used. This indicates that HCH is the limiting substrate in this region and the culture growth is exponential. In our research work, these plots have been used to calculate specific growth rate (*μ*) for that particular initial HCH concentration using Eq. 5. Therefore, the different concentrations of technical HCH ranging from 1 to 50 mg/L were used to determine specific growth rates (Fig. 4); it was observed that the specific growth rate increases with the increase in HCH concentration up to a certain level (10 mg/L). Further, the increase in HCH concentration decreases the specific growth rate. A similar trend has been reported by Lodha et al. (2007) for HCH degradation, where the specific growth rate started decreasing beyond 10 mg/L HCH concentration. The specific growth rates were 0.0011/day and 0.0008 and 0.0012/day at 2 mg/L HCH concentration for DAB-1Y, DAB-1W, and MTCC6362, respectively. The maximum specific growth rate obtained was 0.0089, 0.0068, and 0099/day for DAB-1Y, DAB-1W, and MTCC6362, respectively, at 10 mg/L HCH concentration, beyond that specific growth rate started declining. And was lowest 0.0072 and 0.005 day^−1^ at 50 mg/L for *Kocuria* sp. DAB-1Y and *Staphylococcus* sp. DAB-1W, respectively (data not shown). This suggests that γ-HCH is inhibitory at the higher concentration because it reduces the enzyme activity at higher concentration above 100 mg/L.

### Strain identification

The bacterial cell morphology viz. cell shape, size, color of colony, motility, capsule formation, colony margins etc. was used for the grouping of our two isolates, and biochemical tests were performed to know the metabolic characteristic. The results obtained in this study are presented below.

#### Morphological and biochemical characterization

Gram staining revealed Gram positive, smooth, yellow rods in chains. Positive for citrate utilization, urease, and glucose while negative for lysine, ornithine, TDA, H_2_S production, nitrate reduction, adonitol, lactose, arabinose, and sorbitol. It grows at 8, 25, 45 °C, but not at 4 °C. Also the growth is observed at 1, 5, 7, and 10% NaCl concentration. No growth was observed at 12% NaCl concentration. The pH 7, 8, and 9 was most optimum for growth of the strain *Kocuria* sp. DAB-1Y, while no growth was observed at pH 2, 4, and 11. It showed sporulation and hydrolyzed gelatin and starch. Similar morphological and biochemical tests have been reported by Abhilash et al. (2011) for *Kocuria rhizophila* and by Karn et al. (2011) for *Kocuria* sp. CL2. The *K. rhizophila* colonies reported by Abhilash et al. (2011) were Gram positive, non-motile; showed growth at temperatures 25 to 42 °C, pH 5.2–9, and NaCl 2–7%, hydrolyzed casein and gelatin; showed positive methyl red, catalase tests; and utilized glucose, sucrose, and fructose (Table 3). The *Kocuria* sp. CL2 reported by Karn et al. (2011) was found to be gram positive, coccus, vermillion colored and showed positive oxidase and nitrate reduction tests, while it was negative for catalase, DNAase, indole, urease, and starch hydrolysis tests. *Kocuria varians* reported by Reddy et al. (2003) were gram positive, coccus, and orange and yellow colored, respectively. The *Kocuria rosea* showed positive for catalase, nitrate reduction, and starch hydrolysis, while negative for oxidase, indole, and urease utilization test. The *Kocuria varians* was positive for nitrate reduction and urease test and showed negative for oxidase and starch hydrolysis tests.

**Table 3:** Degradation behavior of lindane isomers by isolates, DAB-1Y and DAB-1W

HCH- isomers	Incubation period (days)	Degradation by isolate DAB-1Y (5 µg/mL) in %	Degradation by isolate. DAB-1W (5 µg/mL) in %
*α*	8	86	93
	28	18	10
*β*	8	12	14
	28	35	32
*γ*	8	94	98
	28	30	28
*δ*	8	80	76
	28	32	28

Data represent mean of three replicates, the MTCC6362 as positive control and flask A (without HCH) as negative control was used in this study

The isolate DAB-1W was found to be Gram negative, white *cocci*, no pigmentation observed, negative for citrate utilization, urease, adonitol, and lactose, and showed positive tests for lysine, ornithine, nitrate reduction, TDA, H_2_S production, glucose, arabinose, and sorbitol. Grows at 25, 45 °C, but not at 4 and 8 °C. Its growth was observed at 1, 5, and 7% NaCl, but not at 10 and 12% NaCl concentration. Grows at pH 5, 7, 8, 9, but not at pH 2, 4, and 11. No sporulation was observed; it hydrolysed starch; and no gelatin hydrolysis was observed (Table 3). Similar results were reported by Abhilash et al. (2011) for *Staphylococcus equorum* and *Staphylococcus cohnii. The S. equorum* was Gram positive, motile; showed growth from 10 to 42 °C temperature, pH 5.2–9, and NaCl concentration 2 to 10%, hydrolyzed citrate; showed positive methyl red, nitrate reduction, and urea tests; and utilized arabinose, glucose, mannose, xylose, sucrose, and fructose. *S. cohnii* was gram positive, non-motile; showed growth from 25 to 42 °C, pH 5.2–9, and NaCl 2 to 10%; showed positive for nitrate reduction, urea tests; and utilized mannose and fructose.

#### Molecular characterization and phylogenetic analysis

**Fig. 5:**
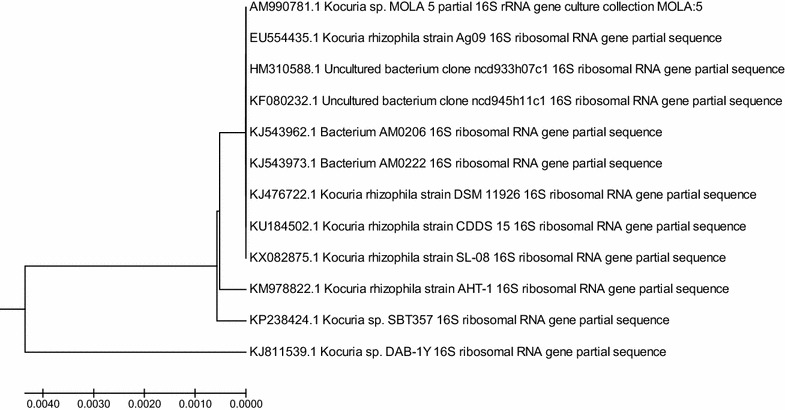
Neighbor-Joining phylogenetic tree of partial 16S rDNA sequence *of Kocuria* sp. DAB-1Y (the query sequence) made using MEGA 7 (Kumar et al. 2016). Evolutionary relationships of *Kocuria* sp. DAB-1Y 16S rRNA gene partial sequence (accession number KJ811539) and the evolutionary history were inferred using the UPGMA method (Sneath and Sokal 1973). The optimal tree with the sum of branch length = 0.00979601 is shown. The tree is drawn to scale, with branch lengths in the same units as those of the evolutionary distances used to infer the phylogenetic tree. The evolutionary distances were computed using the Maximum composite likelihood method (Tamura et al. 2004) and are in the units of the number of base substitutions per site. The analysis involved 12 nucleotide sequences

**Fig. 6:**
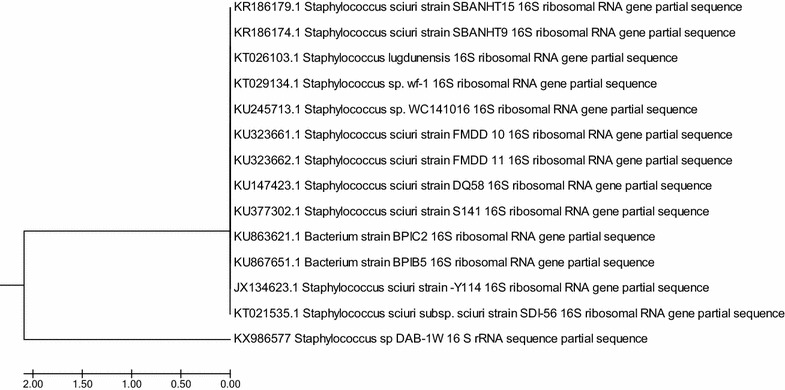
Neighbor-Joining phylogenetic tree of the of partial 16S rDNA sequence of *Staphylococcus* sp. DAB-1w (the query sequence with accession number KX986577) made using MEGA 7 (Kumar et al. 2016), and the evolutionary history was inferred using the UPGMA method (Sneath and Sokal 1973). The optimal tree with the sum of branch length = 4.18104699 is shown. The tree is drawn to scale, with branch lengths in the same units as those of the evolutionary distances used to infer the phylogenetic tree. The evolutionary distances were computed using the Maximum composite likelihood method (Tamura et al. 2004) and are in the units of the number of base substitutions per site. The analysis involved 14 nucleotide sequences

It was observed that the two isolates DAB-1Y and BAB-1W, reported in our study were found to be good candidates for degradation study of HCH, and their strain identification was performed. The DAB-1Y appeared to be a novel strain with significant lindane-degrading activity. Similarly, DAB-1W also shows the good degrading activity. For molecular identification based on 16S rRNA sequencing, the genomic DNA extracted from the isolate DAB-1Y was used to amplify a partial portion (about 965 bp) of 16S rDNA gene. The BLAST analysis of the 16S rDNA sequences suggested the similarity of isolate DAB-1Y to the genus *Kocuria* and was named as *Kocuria* sp. DAB-1Y. The 16S rRNA sequence of it was submitted to NCBI with accession no. KJ811539. The *E* value for all the hits was 0. The evolutionary pattern of the isolate was studied by phylogenetic analysis. A phylogenetic tree of partial 16S rDNA sequence *of Kocuria* sp. DAB-1Y, their selected BLAST hits and previously described γ-HCH isolates were drawn to examine diversity and interrelationships. Similarly, 16S rRNA sequence of DAB-IW showed similarity with *Staphylococcus* sp. by BLAST analysis and it was named as *Staphylococcus* sp. DAB-1W. The evolutionary relationships of *Kocuria* sp. DAB-1Y 16S rRNA and *Staphylococcus* sp. DAB-1W, gene partial sequences (accession number KX986577), and the evolutionary history were inferred using the UPGMA method (Sneath and Sokal 1973), and Evolutionary analyses were conducted in MEGA7 (Kumar et al. 2016). The evolutionary distances were computed using the maximum composite likelihood method (Tamura et al. 2004) and are in the units of the number of base substitutions per site. The phylogenetic analysis of 16S rRNA sequence for *Kocuria* sp. DAB-1Y indicated the three separate clusters and DAB-1Y was found related to these three nodes and found similar with *K. rhizophila* AHT-1, *K. rhizophila* SL08, *Kocuria* sp. SBT357, *K. rhizophila* strain CDDS, *K. rhizophila* DSM11926, Bacterium AM0222, AM0206, *K. rhizophila* Ag09 etc. (Fig. 5). Similarly, the pattern obtained after the phylogenetic analysis of DAB-1W had shown two major clusters (one large and one with bacteria DAB-W our studies), this has shown similarity with 16S rRNA sequence with strains viz. *Staphylococcus sciuri* strain SDI-56, *S. sciuri* strain Y114, Bacterium strain IB5, Bacterium strain BPIC2, *S. sciuri* strain S141, *S. sciuri* strain DQ58, *S. sciuri* strain FMDD 11, *S. sciuri* strain FMDD 10 etc. (Fig. 6).

Potential lindane-degrading microorganisms reported in other research were mostly from genera *Pseudomonas, Sphingobium, Streptomyces, Fusarium* (Salam et al. 2013), and *K. rhizophila, Microbacterium resistens, Staphylococcus equorium, Staphylococcous cohnii subsp. urelyticus* (Abhilash et al. 2011) and *Bacillus brevis* and *Bacillus circulans* isolated from HCH-contaminated soil (Gupta et al. 2000). Salam et al. (2013) isolated a novel yeast strain from sugarcane field, and this has found the ability to use lindane as sole carbon source in the mineral salt medium. The yeast strain was identified and named as *Candida* sp. VITJzN04 based on polyphasic approach which was based on morphological, biochemical, and 18 rDNA sequencing, D1/D2, and ITS sequence analysis. The strain efficiently degraded 600 mg/L of lindane, 6 days of incubation in MSM under optimal conditions (pH 7, temperature 30 °C, and inoculum 0.06 g/L) with half life of 1.17 days and 0.588/day degradation constant. The lindane degradation also tested through kinetic studies as well. Wang et al. (2015) isolated *Arthrobacter nicotianae* DH19 from Ginseng rhizospheric soil using morphological, biochemical tests, and 16S rRNA gene sequencing. This strain utilizes pentachloronitrobenzene (PCNB) as a sole carbon course for growth when it was inoculated in mineral salt medium. It grows at pH 6.85, 30 °C, and inoculum concentration 1.45 g/L and degrade efficiently 90 5 PCNB in 7 days. This strain degrades dichlorodiphenyl trichloroethane, hexachlorocyclohexane, cypermethrin, and cyhalothrin. The metabolites from PCNB degradation were identified using GC–MS. This was first report of PCNB-degrading strain DH19 isolated from rhizospheric soil and therefore, DH19 can be employed in bioremediation of PCNB in the environmental sites cleanup. In the similar study Karn et al. (2011) isolated *Kocuria* sp. CL2 from secondary sludge of pulp and paper mill and this strain was found degradation acitivity against pentachlorophenol (PCP).

**Fig. 7:**
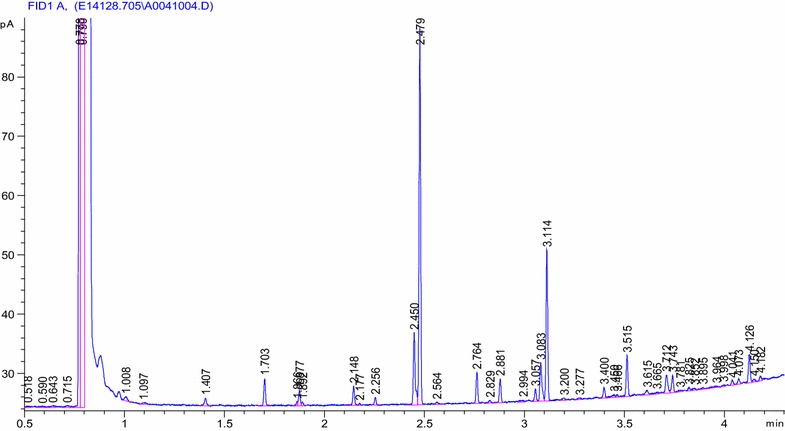
GC-FAME chromatogram of lindane-degrading isolate *Staphylococcus* sp. DAB-1 W showing the characteristic fatty acid peaks

Mainly the strain identification relies on molecular methods like 16S rRNA sequencing, but GC-FAME can also be used. The unique pattern of fatty acids pattern of the strain leads to their identification when compared to already known database. It is a cost-effective technique, the short chain fatty acids produced by the bacterial cell in the standardized media can be used in identification of bacteria. This identifies more than 1500 bacterial species based on their unique fatty acid profiles. The whole cell fatty acids are converted to methyl esters and analyzed by gas chromatography. The fatty acid composition of the unknown organism is compared to a library of known organisms in order to find the closest match. In this study, the lindane-degrading strain DAB-1W was subjected to identification through GC-FAME analysis. But, as compared to GC-FAME, the 16S r RNA-based strain identification is more popular and practiced technique as it is molecular biology-based technique, and leads to accurate species differentiation of species based on their phylogenetic analysis. Slabbinck et al. (2010) discussed the phylogenetic evaluation of 16S rRNA gene data into FAME-based bacterial classification using learning approach called ‘phylogenetic learning.’ They discussed a Supervised Random Forest models for the classification tasks in a stratified cross-validation setting. In this study, we characterized two isolates based on 16 S rRNA-based method, and an additional characterization of DAB-1W strain by GC-FAME was also performed. For the isolate DAB-IW, the 16S rRNA characterization (Fig. 6) and GC-FAME (Fig. 7) was used. These techniques identifies isolate DAB-1W as *Staphylococcus* sp. and named as *Staphylococcus* sp. DAB-IW. The chromatogram shows the peak of retention time (RT) 0.778 corresponds to solvent peak and this was not fatty acid peak. The other peaks of less heights was for the fatty acids detected through GC-FAME technique. Only after this RT, the fatty acids of the isolates started separating and showing various other peaks. Figure 7 and Additional file 1: Tables S1 and S2 indicate the straight chain fatty acid (28%), branched chain fatty acid (41%), MUFA (6.95%), PUFA (17.54%), 18:1 w9c (4.24), 18:2 w6c,9c (2.24), and other minor fatty acids detected (Additional file 1: Tables S1, S2). The fine details of these fatty acids are given in Additional file 1: Table S1.This indicates that SI value in library of RTSBA6 6.21 indicated that SI = 0.526 indicated *Staphylococcus epidermidis*-GC subgroup D (New) and SI = 0.271 shows *Staphylococcus cohnii*. Similar study was conducted by Morey et al. (2013) in which 33 isolates from the Kodiak Seafood Culture Collection (KSCC) and from the American Type Culture Collection (ATCC, USA) were used for GC-FAME-based characterization using Sherlock identification system. The 55% isolates were identified to species with a SI > 0.5 and <0.1, strains of *Pseudomonas fluorescens* and Enterobacteriaceae showed less percentage. They found that saturated straight and branched chain fatty acids were major fatty acids in *Staphylococcus epidermidis* and *Staphylococcus xylosus* but different in quality and quantity. All 5 replicates of *S. xylosus* 12A4 were identified as *S. gallinarum* and it was the only one list in microbial identification system (MIS), but the SI = 0.553 indicates the new species in the chromatogram.

## Conclusion

Lindane is a chlorinated cyclic saturated hydrocarbon used extensively for the control of agricultural pests and mosquitoes. Due to its continuous use in the past decade throughout world, the lindane-contaminated sites are prominent, and thus there is an urgent need to develop its cleanup strategies. Bioremediation is one such technology that could be employed for decontamination of pesticides contaminated soil/sites. Microbial biotechnology possesses ample scope to work in this direction; the new techniques of molecular identification based on high throughput sequencing techniques lead to fine identification of the resistance genes among the strains in less time and cost. In the current work, two novel bacterial strains were isolated which successfully degraded lindane exhibiting 94 and 98% of degrading activity. The results of the present investigation indicate that the strains reported in this study viz *Kocuria* sp. DAB-1Y and *Staphylococcus* sp. DAB-1W may be used as an important biological mean for bioremediation of lindane-contaminated sites.

## Additional file

**Additional file 1: Tables S1, S2.** Sherlock sample report of DAB-1W based on GC-PLFA profiling and the fatty acid profile checked with the standard Sherlock MIDI library gives identification of *Staphylococcus* sp.

## Abbreviations

SI value: similarity index value
MSM: mineral salt medium
HCH: hexachlorocyclohexane
GC-FAME: gas chromatography-fatty acid methyl ester analysis
PCR: polymerase chain reaction
ATCC: American Type Culture Collection, USA
