# Supplementary material for: Degradation study of lindane by novel strains Kocuria sp. DAB-1Y and Staphylococcus sp. DAB-1W
Source: Bioresour Bioprocess. 2016 Dec 28;3(1):53. doi: 10.1186/s40643-016-0130-8 (PMC5196013; doi:10.1186/s40643-016-0130-8)
Supplement: Supplementary file 1 — Additional file 1: Tables S1, S2. Sherlock sample report of DAB-1W based on GC-PLFA profiling and the fatty acid profile checked with the standard Sherlock MIDI library gives identification of Staphylococcus sp. [file 40643_2016_130_MOESM1_ESM.docx]

**Additional files, Table S1**

Volume: DATA File: E141287.05A Samp Ctr: 3 ID Number: 1003

Type: Samp Bottle: 4 Method: RTSBA6

Created: 1/28/2014 5:11:21 PM Library : RTSBA6 6.21

Sample ID: DAB-1W

| RT | Response | Ar/Ht | RFact | ECL | Peak Name | Percent | Comment1 | Comment2 |
| --- | --- | --- | --- | --- | --- | --- | --- | --- |
| 0.2349 | 971 | 0.019 | ---- | 2.9684 |  | ---- | < min rt |  |
| 0.4658 | 841 | 0.020 | ---- | 4.5201 |  | ---- | < min rt |  |
| 0.7877 | 1.349E+9 | 0.018 | ---- | 6.6828 | SOLVENT PEAK | ---- | < min rt |  |
| 1.1030 | 833 | 0.013 | ---- | 8.8013 |  | ---- | < min rt |  |
| 1.2170 | 602 | 0.012 | ---- | 9.5672 | unknown 9.560 | ---- | ECL deviates 0.007 |  |
| 1.4052 | 2269 | 0.011 | ---- | 10.6446 |  | ---- |  |  |
| 1.4758 | 624 | 0.015 | 1.119 | 11.0146 | 11:0 | 0.48 | ECL deviates 0.015 |  |
| 1.7015 | 5930 | 0.009 | 1.061 | 12.0013 | 12:0 | 4.32 | ECL deviates 0.001 | Reference 0.003 |
| 1.8647 | 816 | 0.008 | 1.030 | 12.6209 | 13:0 iso | 0.58 | ECL deviates -0.002 | Reference -0.003 |
| 1.8755 | 3316 | 0.009 | ---- | 12.6621 |  | ---- |  |  |
| 1.8899 | 702 | 0.009 | 1.026 | 12.7166 | 13:0 anteiso | 0.49 | ECL deviates 0.003 | Reference 0.002 |
| 1.9429 | 366 | 0.008 | ---- | 12.9180 |  | ---- |  |  |
| 2.1466 | 4463 | 0.008 | 0.990 | 13.6264 | 14:0 iso | 3.03 | ECL deviates -0.002 | Reference -0.005 |
| 2.2550 | 2430 | 0.011 | 0.977 | 13.9989 | 14:0 | 1.63 | ECL deviates -0.001 | Reference -0.006 |
| 2.4493 | 12315 | 0.009 | 0.959 | 14.6317 | 15:0 iso | 8.10 | ECL deviates 0.000 | Reference -0.007 |
| 2.4606 | 2176 | 0.006 | ---- | 14.6687 |  | ---- |  |  |
| 2.4779 | 59757 | 0.009 | 0.956 | 14.7250 | 15:0 anteiso | 39.19 | ECL deviates 0.000 | Reference -0.006 |
| 2.5624 | 919 | 0.012 | ---- | 14.9998 | 15:0 | ---- | ECL deviates 0.000 |  |
| 2.7631 | 5187 | 0.010 | 0.936 | 15.6336 | 16:0 iso | 3.33 | ECL deviates 0.001 | Reference -0.007 |
| 2.8273 | 999 | 0.010 | 0.932 | 15.8361 | Sum In Feature 3 | 0.64 | ECL deviates -0.004 | 16:1 w7c/16:1 w6c |
| 2.8793 | 4426 | 0.009 | 0.929 | 16.0000 | 16:0 | 2.82 | ECL deviates 0.000 | Reference -0.008 |
| 2.9932 | 493 | 0.009 | ---- | 16.3585 |  | ---- |  |  |
| 3.0552 | 2743 | 0.010 | 0.921 | 16.5534 | 17:1 anteiso w9c | 1.73 | ECL deviates 0.001 |  |
| 3.0816 | 6782 | 0.011 | 0.920 | 16.6365 | 17:0 iso | 4.28 | ECL deviates 0.000 | Reference -0.009 |
| 3.1121 | 18169 | 0.009 | 0.919 | 16.7326 | 17:0 anteiso | 11.45 | ECL deviates 0.000 | Reference -0.009 |
| 3.1965 | 783 | 0.012 | 0.916 | 16.9979 | 17:0 | 0.49 | ECL deviates -0.002 | Reference -0.011 |
| 3.3983 | 1524 | 0.009 | 0.910 | 17.6374 | 18:0 iso | 0.95 | ECL deviates 0.001 | Reference -0.007 |
| 3.4369 | 656 | 0.010 | 0.909 | 17.7595 | Sum In Feature 5 | 0.41 | ECL deviates 0.003 | 18:2 w6,9c/18:0 ante |
| 3.4472 | 563 | 0.007 | 0.909 | 17.7922 | 18:1 w9c | 0.35 | ECL deviates -0.002 |  |
| 3.4638 | 417 | 0.009 | 0.909 | 17.8446 | Sum In Feature 8 | 0.26 | ECL deviates -0.003 | 18:1 w7c |
| 3.5130 | 7602 | 0.010 | 0.908 | 18.0004 | 18:0 | 4.73 | ECL deviates 0.000 | Reference -0.008 |
| 3.6130 | 1706 | 0.014 | ---- | 18.3236 |  | ---- |  |  |
| 3.6636 | 645 | 0.015 | 0.906 | 18.4872 | 19:1 iso I | 0.40 | ECL deviates -0.011 |  |
| 3.7113 | 4288 | 0.012 | 0.905 | 18.6415 | 19:0 iso | 2.66 | ECL deviates 0.003 | Reference -0.004 |
| 3.7412 | 2835 | 0.010 | 0.905 | 18.7380 | 19:0 anteiso | 1.76 | ECL deviates 0.000 | Reference -0.007 |
| 3.7790 | 754 | 0.017 | 0.904 | 18.8601 | Sum In Feature 7 | 0.47 | ECL deviates 0.003 | 19:1 w6c/w7c/19cy |
| 3.8011 | 395 | 0.009 | 0.904 | 18.9315 | 19:0 cyclo w8c | 0.24 | ECL deviates -0.001 |  |
| 3.8230 | 717 | 0.008 | 0.904 | 19.0024 | 19:0 | 0.44 | ECL deviates 0.002 | Reference -0.004 |
| 3.8507 | 827 | 0.015 | ---- | 19.0934 |  | ---- |  |  |
| 3.9578 | 987 | 0.019 | ---- | 19.4463 |  | ---- |  |  |
| 3.9948 | 564 | 0.012 | ---- | 19.5684 |  | ---- |  |  |
| 4.0146 | 376 | 0.009 | 0.903 | 19.6336 | 20:0 iso | 0.23 | ECL deviates -0.002 | Reference -0.008 |
| 4.0396 | 1074 | 0.010 | ---- | 19.7159 |  | ---- |  |  |
| 4.0713 | 1342 | 0.011 | ---- | 19.8203 |  | ---- |  |  |
| 4.0976 | 533 | 0.011 | ---- | 19.9070 |  | ---- |  |  |
| 4.1253 | 7309 | 0.010 | 0.903 | 19.9981 | 20:0 | 4.53 | ECL deviates -0.002 | Reference -0.007 |
| 4.1491 | 897 | 0.011 | ---- | 20.0764 |  | ---- | > max rt |  |
| 4.1811 | 1226 | 0.008 | ---- | 20.1820 |  | ---- | > max rt |  |
| 4.1985 | 455 | 0.010 | ---- | 20.2392 |  | ---- | > max rt |  |
| ---- | 999 | --- | ---- | ---- | Summed Feature 3 | 0.64 | 16:1 w7c/16:1 w6c | 16:1 w6c/16:1 w7c |
| ---- | 656 | --- | ---- | ---- | Summed Feature 5 | 0.41 | 18:0 ante/18:2 w6,9c | 18:2 w6,9c/18:0 ante |
| ---- | 754 | --- | ---- | ---- | Summed Feature 7 | 0.47 | 19:1w7c/19:1 w6c | 19:1 w6c/w7c/19cy |
| ---- | ----- | --- | ---- | ---- |  | ---- | 19:0 cyclo w10c/19w6 |  |
| ---- | 417 | --- | ---- | ---- | Summed Feature 8 | 0.26 | 18:1 w7c | 18:1 w6c |

ECL Deviation: 0.004 Reference ECL Shift: 0.007 Number Reference Peaks: 19

Total Response: 169855 Total Named: 154203

Percent Named: 90.79% Total Amount: 145802

| Matches: | Sim Index | Entry Name |
| --- | --- | --- |
|  | 0.526 | Staphylococcus-epidermidis-GC subgroup D (new strain) |
|  | 0.271 | Staphylococcus-cohnii-cohnii |

**Table S2**

Volume: DATA File: E141294.FAT Samp Ctr: 4 ID Number: 1003

Type: Samp Bottle: 0 Method: FATYPE

Created: 1/29/2014 12:23:19 PM

Sample ID: DAB-1W

Operator Comment: System 1/29/2014 12:23:19 PM

Categorize based on file C:\SHERLOCK\EXE\PLFA\PLFAD1toFA.txt

| RT | Response | Ar/Ht | RFact | ECL | Peak Name | Percent | Comment1 | Comment2 |
| --- | --- | --- | --- | --- | --- | --- | --- | --- |
| 1.000 | 73624 | 0.100 | 1.000 | 1.000 | Straight | 28.16 | ECL deviates 0.000 |  |
| 2.000 | 106878 | 0.100 | 1.000 | 2.000 | Branched | 40.88 | ECL deviates 0.000 |  |
| 5.000 | 18169 | 0.100 | 1.000 | 5.000 | MUFA | 6.95 | ECL deviates 0.000 |  |
| 6.000 | 45067 | 0.100 | 1.000 | 6.000 | PUFA | 17.54 | ECL deviates 0.000 |  |
| 9.000 | 11074 | 0.100 | 1.000 | 9.000 | 18:1 w9c | 4.24 | ECL deviates 0.000 |  |
| 10.000 | 5865 | 0.100 | 1.000 | 10.000 | 18:2 w6,9c | 2.24 | ECL deviates 0.000 |  |
| 12.000 | 1398 | 0.100 | ---- | 12.000 | Other | ---- | ECL deviates 0.000 |  |

ECL Deviation: 0.000 Reference ECL Shift: 0.000 Number Reference Peaks: 0

Total Response: 261453 Total Named: 261453

Percent Named: 100.00% Total Amount: 261453

(No search libraries specified in method FATYPE.)
